# Investigation of three common centrifugation protocols for platelet-rich fibrin (PRF) as a bio-carrier for ampicillin/sulbactam: a prospective trial

**DOI:** 10.1007/s00784-023-05212-x

**Published:** 2023-08-21

**Authors:** Anton Straub, Chiara Utz, Maximilian Stapf, Andreas Vollmer, Sylvia Kasper, Alexander C. Kübler, Roman C. Brands, Stefan Hartmann, Thiên-Trí Lâm

**Affiliations:** 1 Department of Oral and Maxillofacial Plastic Surgery of the University Hospital Würzburg, Pleicherwall 2, 97070 Würzburg, Germany; 2 Institute for Pharmacy and Food Chemistry of the University of Würzburg, Am Hubland, 97074 Würzburg, Germany; 3 Institute for Hygiene and Microbiology of the University of Würzburg, Josef-Schneider-Street 2/E1, 97080 Würzburg, Germany

**Keywords:** Platelet-rich fibrin, PRF, Bio-carrier, Ampicillin, Sulbactam, Antibiotics, Agar diffusion test, Local antibiotic application, Centrifugation, Protocol

## Abstract

**Objectives:**

Different platelet-rich fibrin (PRF) protocols exist and are known to differ in resulting mechanical and bioactive properties. Centrifugation parameters may also influence drug release, in particular antibiotics, when using PRF as a bio-carrier. We thus evaluated three common protocols regarding effects on the bio-carrier properties.

**Materials and methods:**

In a prospective trial comprising 33 patients, we compared different protocols for PRF as a bio-carrier for ampicillin/sulbactam (SAM). Blood samples were taken shortly after a single dose of ampicillin/sulbactam (2 g/1 g) was administered to patients intravenously. PRF was obtained by centrifugation and three protocols were used: protocol A (1300 rpm, 8 min, RCF-max = 208 g), B (2300 rpm, 12 min, RCF-max = 652 g), and C (1500 rpm, 14 min, RCF-max = 276 g). The antibacterial activity of PRF was investigated against five oral species in vitro, based on agar diffusion methodology.

**Results:**

The study demonstrates that a single dose of SAM is sufficient to reach high concentrations in PRF in all protocols (150 µg/ml), which is comparable to the plasma SAM concentration. Antibacterial activity was inferred from the diameter of inhibition zones seen in agar diffusion tests using PRF discs. Protocol B resulted in the largest inhibition zones. One-way ANOVA revealed statistically improved results for protocol B for some bacteria.

**Conclusions:**

The study provides valuable data on PRF antibiotic enrichment, notably SAM. A single dose of SAM is sufficient to reach clinically relevant concentrations in PRF.

**Clinical relevance:**

These findings potentially extend the application of PRF, for example in patients with osteonecrosis of the jaw or in oral surgery (e.g., stick bone).

## Introduction

Platelet-rich fibrin (PRF) is a blood product widely used in oral and maxillofacial surgery, which is acquired by centrifuging whole blood samples of a patient [1]. PRF does not contain further additives such as anticoagulants [2]. It is widely accepted that the release of growth factors from PRF supports and improves wound healing [1, 3–6]. Among others, the release of platelet-derived growth factor (PDGF), transforming growth factor (TGF)-beta, and interleukins modulates the immune response, as well as cell stimulation and differentiation. Vascular endothelial growth factor (VEGF), on the other hand, increases angiogenesis [1, 2]. In addition to such bioactive properties, PRF can also be used to improve the mechanical properties for example of bone or bone substitute material when it is mixed with injectable PRF (sticky bone) [7]. In osteonecrosis of the jaw (ONJ), solid PRF membranes can serve as an additional saliva-proof seal and a lubrication layer. Furthermore, the membranes can cover sharp bone edges [8, 9].

The implementation of PRF as a carrier for drugs has already been described [10]. Among others, the suitability of PRF as a bio-carrier for antibiotics has been investigated. Antibiotics for local application via PRF were either administered systemically to the patient, or added to the blood sample before or after centrifugation [8, 11, 12]. In a previous study, we demonstrated that intravenously administered ampicillin/sulbactam (SAM) substantially accumulates in PRF products and is released from the membranes in antimicrobially effective concentrations over periods of several days [8]. Other studies have revealed PRF to possess intrinsic antimicrobial properties without the addition of antibiotics [13–15].

Several centrifugation protocols for PRF have been developed, resulting in different bioactive and mechanical properties [4, 5, 16–18]. The ensuing PRF products differ in fibrin structure and tensile strength [19]. Reducing the centrifugation speed and time leads to an increase in the number of cells in the PRF product [20, 21] resulting in a significantly greater release of growth factors [4, 5, 16].

Protocols may also differ in regard to the PRF quality as a bio-carrier for drugs, which has never been investigated before. Therefore, the objective of this study was to evaluate solid PRF as bio-carrier of antibiotics obtained according to three common PRF protocols [4, 16–18]. We hypothesized that the differences in fibrin structure not only influence the mechanical properties and the release of growth factors, but also the release of drugs and antibiotics, in particular SAM. Furthermore, we explored whether an intravenous single dose of SAM is sufficient to achieve effective antibiotic concentrations in the PRF membranes and whether the antibiotic activity of PRF correlates with the time interval between infusion and blood sampling to obtain PRF.

## Methods

**Fig. 1:**
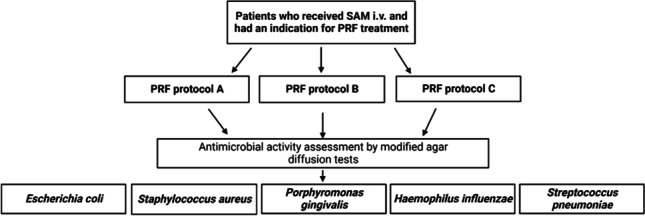
Flowchart: Patients undergoing antibiotic therapy with SAM and indicated as requiring PRF application to treat a disease in the maxillofacial region were included. Upon blood sampling, PRF was obtained according to one of three different protocols (see “PRF protocols”). Agar diffusion tests were performed with the PRF membranes resulting from the three protocols on the five indicated oral bacteria. Abbreviations: PRF, platelet-rich fibrin; SAM, ampicillin/sulbactam; i.v., intravenously

In this prospective trial, blood was sampled from a total of 33 adults. All participants were patients of the University Hospital Würzburg between September and December 2022 (Fig. 1). We set the study inclusion criteria to be an age of 18 or over, the indication for first-dose antibiotic prophylaxis or treatment with SAM (Unacid®, Pfizer Pharma GmbH, Berlin, Germany, 3g) that we actually administered, and the application of PRF to a surgical site. Patients were excluded if they were taking any antibiotic treatment or prophylaxis other than the single dose of SAM we administered. We also excluded patients if there was any failure to comply with protocols in the study after being included, such as errors in blood sampling, or blood and PRF storage times being incorrect or exceeded. The institutional review board of the University of Würzburg approved all the protocols implemented in this study (IRB approval number: 143/20-me).

### Blood sampling and antibiotic therapy

Patients received a single dose of SAM before the surgical intervention as perioperative prophylaxis (e.g., fractures, ONJ, see Fig. 1) or as part of an infection therapy protocol/regimen. Blood sampling for PRF was always performed promptly after the first intravenous administration of 2 g/1 g SAM intraoperatively. For PRF blood sampling, depending on the specific surgical intervention, four to six sterile 10 ml vacuum glass A-PRF tubes (Process for PRF, Nice, France) were used. For all protocols and patients, the same PRF tubes were used. PRF was obtained with a Duo Quattro centrifuge (Process for PRF, Nice, France) implementing one of the three different protocols (see “PRF protocols”). Blood clots were pressed and PRF membranes were fabricated in the usual manner [22, 23].

### PRF protocols

We implemented three common protocols for PRF production with a Duo Quattro centrifuge, thereby adjusting the centrifugation parameters and taking into account the angulation of the tubes in the centrifuge to match the centrifugation parameters. PRF in all protocols was produced by fixed-angle centrifugation. In protocol A, we followed a preformed protocol provided by the Duo Quattro centrifuge manufacturer (Advanced PRF plus (A-PRF +), 1300 rpm for 8 min, RCF-max = 208 g) [17]. For protocol B, in which centrifugation was originally at 2700 rpm (RCF-max = 653 g) and 12 min with an Intraspin® centrifuge (Intralock International, Birmingham, USA), we reduced the centrifugation force with the DuoQuattro centrifuge to 2300 rpm (RCF-max = 652 g) owing to the different angulation of the tubes [4, 17, 18]. In protocol C, we followed the standard advanced PRF protocol (A-PRF) provided by the manufacturer (1500 rpm, 14 min, RCF-max = 276 g) [5, 17]. Centrifugation was performed using a rotor angulation of 40° with a radius of 77 mm at the clot and 110 mm at the maximum) [24].

### Ampicillin/sulbactam concentration

**Fig. 2:**
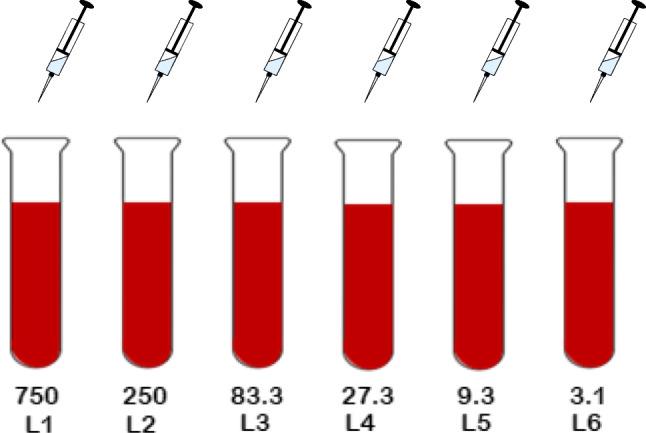
PRF preparation with defined SAM concentrations for linear regression analyses: various concentrations 750, 250, 83.3, 27.8, 9.3, and 3.1 µg/ml ampicillin and a fixed concentration of 50 µg/ml sulbactam for L1-6. Blood was subsequently centrifuged with protocol A (1300 rpm, 8 min). Abbreviations: L, line

To estimate the SAM concentration in the PRF membranes, PRF was prepared with different known ampicillin concentrations and a linear regression analysis was performed. Six different ampicillin concentrations were combined respectively with a fixed sulbactam concentration (50 µg/ml, see Fig. 2). Blood for PRF preparation from patient controls without antibiotic therapy was also collected as described above. From an ampicillin stock solution (75 mg/ml), aqueous working solutions were prepared to obtain the following concentrations: 75 mg/ml, 25 mg/ml, 8.33 mg/ml, 2.78 mg/ml, 0.93 mg/ml, and 0.31 mg/ml. Of each of these aqueous solutions, 100 µl was added to 10 ml of blood, resulting in the following concentrations: 750 µg/ml, 250 µg/ml, 83.3 µg/ml, 27.8 µg/ml, 9.3 µg/ml, and 3.1 µg/ml. Furthermore, 100 µl of a sulbactam stock solution (5 mg/ml) was added to the blood before centrifugation (50 µg/ml sulbactam in the blood sample). The blood was then centrifuged according to PRF protocol A (1300 rpm, 8 min) and placed on an agar plate inoculated with *Streptococcus pneumoniae* ATCC 49619. The inhibition zones were measured after 24 h. The same experiments were carried out with PRF discs stored at 36 °C for 24 h before being placed on the inoculated plates for a further 24 h. From the data, a calibration curve was created so that the SAM concentrations in the PRF membranes could be determined approximately from the measured inhibition zones of the described experiments.

### Agar diffusion tests

Modified agar diffusion tests were performed based on the EUCAST disc agar diffusion methodology with *Haemophilus influenzae* ATCC 49766; *Staphylococcus aureus* ATCC 29213; *Streptococcus pneumoniae* ATCC 49619; and *Escherichia coli* UR-6454–201/2022, a clinical isolate from a urine sample; and *Porphyromonas gingivalis* ATCC 33277 isolated from a human throat [25]. As there are no testing guidelines from EUCAST for *P. gingivalis*, preliminary tests were performed to establish a suitable test protocol based on procedures according to EUCAST. Bacterial suspensions for *H. influenzae, S. aureus, S. pneumoniae,* and *E. coli* were adjusted to a McFarland turbidity standard of 0.5 in 0.85% sodium chloride (w/v) in water using a DensiCHEK Plus (bioMérieux, Nürtingen, Germany). The bacterial suspension for *P. gingivalis* was adjusted to a McFarland turbidity standard of 1.0 using the same procedure. *S. aureus* and *E. coli* inocula were plated on unsupplemented Mueller–Hinton E agar (MH-E, bioMérieux, Nürtingen, Germany), *S. pneumoniae* and *H. influenzae* were plated on Mueller–Hinton agar containing 5% defibrinated horse blood and 20 mg/l β-NAD (MH-F, BD, Heidelberg, Germany). *P. gingivalis* was plated on Brucella Blood Agar with Hemin and Vitamin K1 (BD Brucella Blood Agar with Hemin and Vitamin K1, Heidelberg, Germany).The McFarland-adjusted bacterial suspension was spread evenly over the entire surface of the agar plate using a cotton swab. A 6mm PRF disc (produced using protocol A, B, or C) was placed on each inoculated plate and incubated for 24 h. We performed eleven experiments for each protocol and bacterium. The same experiments were carried out with a 6 mm PRF disc stored at 36 °C for 24 h before being placed on the inoculated plates for a further 24 h.

**Fig. 3:**
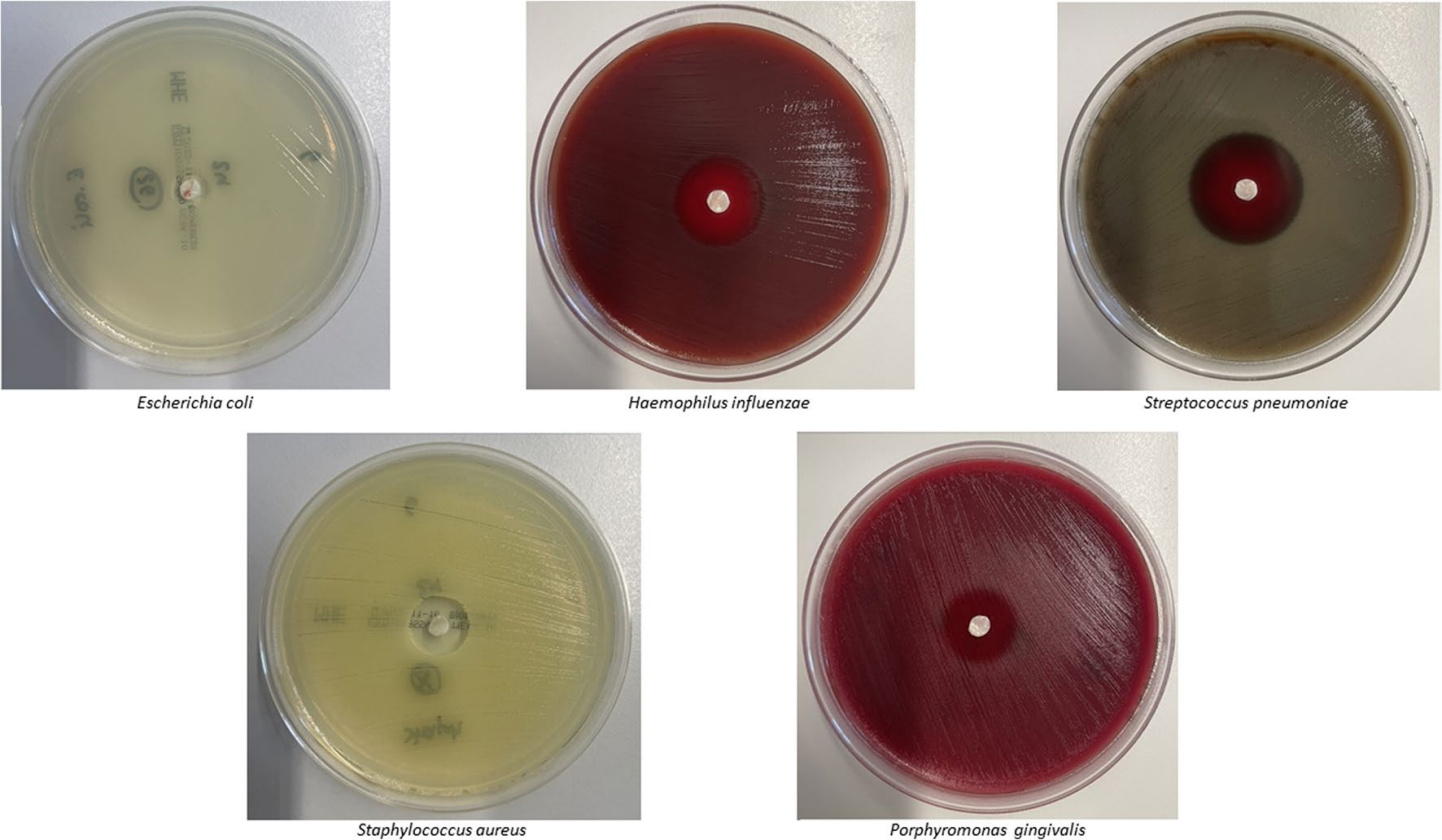
Agar diffusion test with 6mm PRF discs (protocol A) after infusion of 3 g SAM and incubation for 24 h

We employed a number of technical controls. Firstly, a disc agar diffusion test was performed in parallel using an antimicrobial susceptibility test disc (ThermoScientific Oxoid, Langenselbold, Germany) loaded with 20 µg ampicillin/sulbactam. Secondly, a gradient agar diffusion test was completed using a test strip (Liofilchem, Roseto degli Abruzzi, Italy) loaded with an ampicillin gradient ranging from 0.016 to 256 mg/l and a fixed sulbactam load of 4mg/l (Fig. 3). Agar plates were incubated at 36 °C for 24 h in ambient air (*S. aureus*, *E. coli*) or at 35 °C in 5% CO_2_ atmosphere (*H. influenzae* and *S. pneumoniae*). Agar plates containing *P. gingivalis* were stored in an anaerobic box with a GENbox anaer bag (bioMérieux, Nürtingen, Germany) and Dry Anaerobic Indicator Strips BD-BBL (BD, Heidelberg, Germany) for seven days at 36°C until read-out was completed. Upon incubation, the diameters of the inhibition zones were measured in millimetres and photographs were taken for documentation purposes (Fig. 3).

### Statistics

Statistical analysis was performed using Prism Version 9 (GraphPad Software Inc., San Diego, La Jolla, USA). Differences between the means were examined using one-way analyses of variance (ANOVA, *α* = 0.05) and considered significant if *p*-values were less than 0.05 (*p < 0.05). Any correlation between time of infusion and blood sampling with the diameter of the inhibition zone (IZ) in the agar diffusion test was determined with the Pearson-test. Descriptive statistics were also analysed with Prism Version 9 (GraphPad Software Inc., San Diego, La Jolla, USA).The resulting data is expressed as means and standard deviations.

## Results

A total of 33 patients with a mean age of 54 years were included in the trial comprising 26 males and 7 females (see Table 1). PRF blood sampling was performed at a mean time of 16.9 ± 9.7 min after the infusion of SAM.

**Table 1:** Descriptive statistics

	Participants
*N* (total)	33
Male/female ratio	26/7
Mean age (in years)	54 (SD ± 20.6)
Age range	18–85
Diagnosis	
ONJ	7
Infection	6
Malignoma	9
Cysts	3
Orthognathic surgery	2
Traumatology	2
Tooth extraction	4
Time after infusion (min)	16.9
Renal function (MD ± SD):	88.5 (SD ± 20.9) ml/min*

*N* number of participants, *ONJ* osteonecrosis of the jaw

*Glomerular filtration rate (MDRD) in ml/min

### Fresh PRF

After incubation for 24 h, the PRF discs obtained following protocol B (2300 rpm, 12 min) resulted in the largest inhibition zones, compared to the PRF discs from protocols A and C (1300 rpm, 8 min; 1500 rpm, 14 min, see Table 2). The sizes of the inhibition zones for *E. coli* were found to be statistically significant with respect to the protocols (one-way ANOVA; *p* = 0.04). For *S. aureus*, *S. pneumoniae*, *H. influenzae*, and *P. gingivalis,* inhibition zone diameters for PRF discs obtained by the three protocols were found not to be statistically significant (Table 2).

**Table 2:** Average inhibition zones of 6 mm PRF discs with various centrifugation parameters after 24 h of incubation (diameter in mm)

	*Escherichia coli*	*Staphylococcus aureus*	*Streptococcus pneumoniae*	*Haemophilus influenzae*	*Porphyromonas gingivalis*
1300 rpm, 8 min	11.2	17.2	23.3	19.6	13.9
2300 rpm, 12 min	12.5*	17.9	23.9	22.1	21.4
1500 rpm, 14 min	10.1	16.6	23.55	20.5	19.8

*Statistically significant result

### PRF after storage for 24 h

We observed similar results even if the PRF discs were stored for 24 h at 36 °C prior to testing. PRF discs obtained from protocol B also caused the largest inhibition zones compared to the discs from protocols A and C (Table 3). However, the diameters of the inhibition zones were smaller than those observed in PRF discs that had not been stored, and the inhibition zones differed less in size between the protocols. One-way ANOVA was significant for *E. coli*, *H. influenzae,* and *P. gingivalis* (*p* = 0.04, 0.03, and 0.02, respectively, Table 3).

**Table 3:** Average inhibition zones of 6 mm PRF discs with various centrifugation parameters after storage for 24 h (diameter in mm)

	*Escherichia coli*	*Staphylococcus aureus*	*Streptococcus pneumoniae*	*Haemophilus influenzae*	*Porphyromonas gingivalis*
1300 rpm, 8 min	6.5	13.6	20.5	14	6.3
2300 rpm, 12 min	7.9*	14.7	21.1	18.1*	14.7*
1500 rpm, 14 min	4.0	13.7	19.9	14.2	9.6

*Statistically significant result

### Correlation between time of blood sampling and the size of the inhibition zone

On average, blood for the PRF production was sampled approximately 17 min after the SAM infusion. A positive correlation between the time period between infusion and blood sampling and the diameter of the inhibition zones was found for *S. aureus* and *S. pneumoniae* using Pearson’s test (*p* = 0.01 and 0.0006 and *r* =  − 0.4 and − 0.6, see Table 4).

**Table 4:** Correlation between time of blood sampling and the size of the inhibition zone

	*Escherichia coli*	*Staphylococcus aureus*	*Streptococcus pneumoniae*	*Haemophilus influenzae*	*Porphyromonas gingivalis*
*p* value*	0.25	0.01	0.0006	0.4	0.9
Correlation coefficient (*r*)	− 0.2	− 0.4	− 0.6	− 0.1	0.02

*One-way ANOVA

### Antibiotic concentration in PRF

**Fig. 4:**
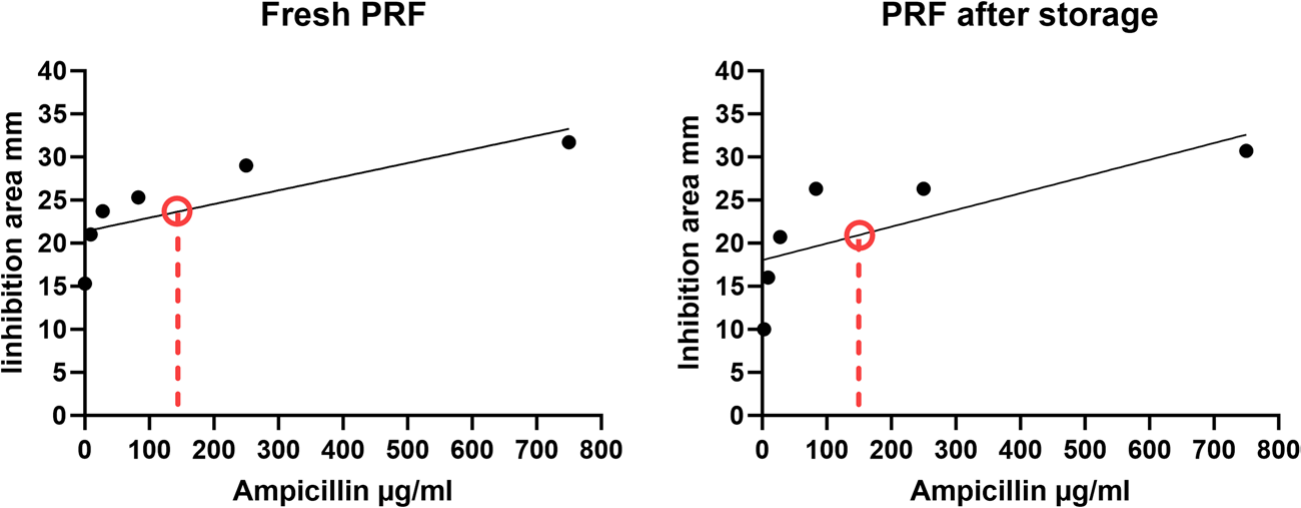
Linear regression after incubation 24 h with fresh PRF (left) and PRF stored for 24 h (right). As portrayed in the figure, the estimated antibiotic load in a PRF membrane is 147 µg/ml (fresh PRF) and 155 µg/ml (PRF after storage)

To determine the mean antibiotic load of a PRF disc, PRF was produced (protocol A, 1300 rpm, 8 min) with various concentrations of ampicillin (six concentrations from 750 to 3.1 µg/ml) and a fixed concentration of sulbactam (50 µg/ml). A *S. pneumoniae* agar plate was subsequently incubated with the PRF discs directly (fresh PRF) or after storage for 24 h (PRF after storage). The inhibition zone was measured after incubation for 24 h as described above. Regression analysis revealed a correlation to the given antibiotic concentration and the inhibition zone for both fresh and stored PRF (see Fig. 4). With a mean inhibition zone of 23.3 mm and 20.5 mm (fresh PRF and PRF after storage, respectively, *S. pneumoniae*, protocol A at 1300 rpm and 8 min), the SAM concentration in the PRF discs was estimated to be 146.6 µg/ml (fresh PRF) and 154.7 µg/ml (PRF after storage). We determined no significant difference between the fresh and stored PRF time points (*t*-test, *p* = 0.5, see Fig. 4).

## Discussion

This study evaluated the quality of different PRF protocols to produce PRF as bio-carriers for antibiotics. It has previously been demonstrated that PRF may be used as a carrier and matrix for drugs, which were released from the PRF [10]. Furthermore, several studies have revealed that the bioactive properties (release of growth factors and antimicrobial effects) as well as the mechanical properties, such as fibrinogen concentration and tensile strength, were influenced by the centrifugation parameters, notably centrifugation speed and time [4, 15–17, 20, 26]. To our knowledge, this is the first investigation into the influence of the centrifugation parameters on the antimicrobial activity of PRF.

We examined three common protocols used in the preparation of PRF for their properties as bio-carriers of SAM. The same plain glass tube for PRF production were used for all protocols. This seems important as it is reported that PRF membranes depend on centrifugation tubes. Especially silica-coated plastic tubes could have some negative aspects like contamination of the PRF product with cytotoxic silica microparticles [27]. We implemented a modified agar diffusion method based on EUCAST with the PRF discs obtained using these three protocols and five different oral bacteria. Statistically significantly greater antibacterial activity towards *E. coli* was observed for protocol B (2300 rpm, 12 min, RCF-max = 652 g) with fresh PRF. We also tried to simulate the in vivo situation by storing the PRF discs at 36 °C for 24 h to investigate this longer-term effect. Here, our data reveal an even greater advantage in favour of protocol B with a significantly improved antimicrobial effect on more bacterial species. Our data thus suggest that the use of this protocol should be favoured if local antibiotic treatment is the main goal of the PRF application. We demonstrated in a previous study that the antibiotic concentration in PRF is comparable to the plasma antibiotic concentration when patients received at least three infusions of SAM [8]. All of these patients suffered from osteonecrosis of the jaw, either following radiation therapy of the head and neck area or after administration of antiresorptive drugs such as bisphosphonates or denosumab. However, it remained unclear as to how many infusions are necessary at minimum to achieve sufficient concentrations of SAM in the PRF product. Furthermore, it was uncertain whether there is any effect of the disease patients present on PRF as a product and its function as a drug carrier. In this study, the participants were not selected according to the presented disease and received only one dose of SAM (see Table 1 for the various diagnoses). We estimated the SAM concentration in PRF to be 150 µg/ml, which is in line with the plasma concentration reported in the literature [8, 28–32]. Furthermore, the concentration in PRF after at least three infusions of SAM is comparable to our calculated SAM concentration [8]. With a single dose of 2/1 g SAM, the minimum inhibitory concentration was exceeded for *S. aureus, S. pneumoniae, H. influenzae, P. gingivalis,* and *E. coli* [33]. These bacteria represent species known to cause infections in the oral cavity [34]. Taken together, our data demonstrate that a single dose of SAM is sufficient to achieve therapeutic concentrations in the PRF. Our results also suggest that the underlying disease does not affect the properties of PRF as a bio-carrier.

A high correlation between the time period between infusion and blood sampling with the plasma antibiotic concentration has already been described [30, 31, 35]. Wildfeuer et al. measured for example a SAM plasma concentration of 97/37.6 µg/ml 30 min after infusion. In another study, a SAM plasma concentration of 124.9/96.5 µg/ml was measured 20 min after infusion [32]. In our study, the mean time gap between infusion and blood sampling for PRF was approximately 17 min. Accordingly, we found a correlation between the time period between infusion and blood sampling and the diameter of the inhibition zones in the modified agar diffusion tests. Furthermore, the diameter of the inhibition zones correlated with the antibiotic concentration in the PRF products. Therefore, we conclude that blood sampling for PRF should be performed after a short distribution period post infusion, to ensure the highest possible concentration in the PRF product. This is in line with the result that the SAM concentrations in PRF we measured approximately 17 min after SAM infusion are slightly higher than plasma SAM concentrations reported in the literature [8, 32, 35].

So far, no study has investigated how long SAM is stable in PRF products, with which dynamic it is released, and whether it has any antibacterial effect. We have now been able to demonstrate that SAM is released from PRF, even when stored for 24 h at 36 °C. Further studies will be necessary to investigate how long the effect lasts beyond that. This could have enormous clinical benefit, for example, in the treatment of patients with osteomyelitis or ONJ undergoing prolonged systemic antibiotic therapy [36]. In oral surgery, for example, such antibiotic-enriched PRF could prove useful for local antibiotic application in bone augmentations with autologous bone or allogenic and xenogeneic bone-substitute material, in sticky bone, and other procedures [37].

A limitation of this study is the lack of data on how other commonly used antibiotics accumulate in PRF and are released. Investigation of the long-term release of SAM would be particularly crucial regarding the indication of PRF application. It is however already remarkable that we were able to demonstrate that SAM is stable in PRF for more than 48 h, although the half-life of ampicillin as well as sulbactam is only one to two hours [38]. PRF seems to extend the half-lives, which underscores its function as a bio-carrier. In some circumstances, this could lead to dose reductions in systemically administered antibiotics, as is the case in patients with ONJ or osteomyelitis. Further studies should therefore focus on investigating different antibiotics and different administration options (oral versus intravenous). We investigated the in vitro antibacterial effect of PRF in monocultures. However, most bacteria in the oral cavity grow in biofilms, which would require higher minimal inhibitory concentrations for treatment [39]. Further studies should therefore investigate whether the antibacterial effects of antibiotic-enriched PRF are sufficient to penetrate oral biofilms, thus preventing and fighting infections in vivo. In the presented study, PRF was produced by fixed-angle centrifugation in all protocols. Recent research demonstrated that horizontal centrifugation optimizes cell distribution and leads to a higher cell count of leukocytes in PRF compared to fixed-angle centrifugation [40]. Therefore, it is possible that the centrifugation process (fixed-angle versus horizontal) influences the carrier properties of PRF, which should be investigated by further research.

## Conclusion

The study presented here provides valuable information on how PRF can be enriched with antibiotics, notably SAM. We demonstrate especially that a single dose of SAM is sufficient to reach high concentrations within PRF when the sampled blood is obtained no longer than 15 min after infusion of SAM. These results are clinically relevant and have the potential to expand the field of PRF application. Nevertheless, complementary studies will be necessary to investigate the long-term effect, the release kinetics, and the behaviour of different antibiotics in PRF.

## Data Availability

The dataset supporting the conclusions of this article is included within the article. Further clinical data and information are not publicly available because other, currently unpublished studies are based on it, but are available from the corresponding author on reasonable request.

## Abbreviations

*MIC*: Minimum inhibitory concentration
*ONJ*: Osteonecrosis of the jaw
*PDGF*: Platelet-derived growth factor
*PRF*: Platelet-rich fibrin
*SAM*: Ampicillin/sulbactam
*TGF*: Transforming growth factor
*VEGF*: Vascular endothelial growth factor
